# Role of Sox-9, ER81 and VE-Cadherin in Retinoic Acid-Mediated Trans-Differentiation of Breast Cancer Cells

**DOI:** 10.1371/journal.pone.0002714

**Published:** 2008-07-16

**Authors:** Yoshimi Endo, Kamla Deonauth, Priya Prahalad, Becky Hoxter, Yuelin Zhu, Stephen W. Byers

**Affiliations:** 1 Lombardi Comprehensive Cancer Center, Department of Oncology, Georgetown University, Washington D. C., United States of America; 2 Department of Biology, Howard University, Washington D. C., United States of America; Dresden University of Technology, Germany

## Abstract

Many aspects of development, tumor growth and metastasis depend upon the provision of an adequate vasculature. This can be a result of regulated angiogenesis, recruitment of circulating endothelial progenitors and/or vascular trans-differentiation. The present study demonstrates that treatment of SKBR-3 breast cancer cells with retinoic acid (RA), an important regulator of embryogenesis, cancer and other diseases, stimulates the formation of networks in Matrigel. RA-treatment of SKBR-3 cells co-cultured with human umbilical vein endothelial cells resulted in the formation of mixed structures. RA induces expression of many endothelial genes including vascular endothelial (VE) cadherin. VE-cadherin was also induced by RA in a number of other breast cancer cells. We show that RA-induced VE-cadherin is responsible for the RA-induced morphological changes. RA rapidly induced the expression of Sox-9 and ER81, which in turn form a complex on the VE-cadherin promoter and are required to mediate the transcriptional regulation of VE-cadherin by RA. These data indicate that RA may promote the expression of endothelial genes resulting in endothelial-like differentiation, or provide a mechanism whereby circulating endothelial progenitor cells could be incorporated into a growing organ or tumor.

## Introduction

Organogenesis during embryonic development and tumor growth are dependent on adequate vasculature. For example a breast tumor that is unable to induce angiogenesis fails to reach a size greater than 4 mm and is incapable of distant metastasis. Metastatic lesions are often the source of cancer symptoms and the primary cause of death in cancer patients. Thus, the degree of vascularization of the tumor is one of the most important negative prognostic parameters in breast cancer.

Illustrating how a tumor induces new vasculature is, therefore, a crucial step to understand molecular nature of tumor progression. The classic paradigm of the angiogenic switch states that, as the tumor expands, central necrosis occurs due to hypoxia and nutrient deprivation[1]. This leads to the production of angiogenic factors such as VEGF and the recruitment of endothelial cells from neighboring blood vessels or circulating progenitors[2]. An alternative concept of tumor angiogenesis has been proposed in which some tumor cells trans-differentiate into endothelial cells and give rise to patterned spheroidal tumor clusters containing channel-like spaces between them capable of mimicking the circulatory system [3]. This phenomenon, termed as “vasculogenic mimicry”, is highly predicative of clinical outcome in patients with aggressive melanoma. Vasculogenic mimicry also occurs in other tumor types including breast cancer [4]–[7]. In the breast cancer model, blood flows between tumor cell-lined vascular spaces and endothelium-lined and/or mature vasculature. However, detailed molecular mechanisms underlying vasculogenic mimicry still remain to be elucidated, and it is not known if any treatment regimens can regulate this process.

Retinoids, natural or synthetic vitamin A analogues, have been used in anti-cancer therapy for many years. RA exhibits differentiation and cell cycle arrest-dependent growth inhibition properties in many cells and tissues. Many clinical and experimental model studies have shown that retinoids and their derivatives are effective in prevention and treatment of breast cancer [8]–[10]. On the other hand, treatment of primary lung cancer with β-carotene, the most important dietary precursor of vitamin A, induced an even higher incidence of lung cancer [11], [12]. Fenretinide, a vitamin A analogue, appears to reduce the risk of second breast cancers in premenopausal women, but increases risk in postmenopausal women [13]. These studies imply that retinoids may be effective in a preventative setting, but may actually have a negative effect after tumor initiation and during progression, particularly in estrogen-depleted circumstances. As some tumor cells may harbor a pluripotent stem cell phenotype, retinoid-induced “differentiation” during carcinogenesis could result in either a negative outcome (e.g. vascular or mesenchymal differentiation) or a positive one (e.g. mammary epithelial differentiation). In other words, retinoid-dependent “differentiation” may be responsible both for its failure as a treatment in certain circumstances as well as its side effects, due to its action on non-tumor cells. In the present study, we report that 9-*cis*-RA induced endothelial-like differentiation in the estrogen receptor negative SKBR-3 breast adenocarcinoma cell line. Using microarray analysis, we found that 9-*cis*-RA treatment resulted in an endothelial-like genetic program and robustly induced VE-cadherin. 9-*cis*-RA treatment induced VE-cadherin in four other breast cancer cells. We also show that Sox-9 and the Ets-1 family member ER81 are required for the transcriptional regulation of VE-cadherin by 9-*cis*-RA.

## Materials and Methods

### Materials and Reagents

9-*cis*-RA was obtained from Sigma-Aldrich (Germany), mouse anti-VE-cadherin was from Immunotech (France) and R&D systems (Minneapolis, MN), goat anti-VE-cadherin were from Santa Cruz Biotechnology (Santa Cruz, CA), rabbit anti-Sox-9 was from Chemicon (Temecula, CA), anti-myc was from Invitrogen (Carlsbad, CA), anti-FLAG (M2) was from Sigma, anti-GAPDH was from Research Diagnostics Inc (Flanders, NJ). Small interference RNA (siRNA) reagent (SMART pool) for human VE-cadherin was purchased from Dharmacon (Lafayette, CO). As a control siRNA, Luc siRNA was used (CGUACGCGGAAUACUUCGA). Plasmid DNA encoding wild type (WT) Sox-9 and dominant negative (DN) form of Sox-9 (lacking the C-terminal trans-activation domain) were kindly provided by Dr. Véronique Lefebvre (Cleveland Clinic Foundation, Cleveland, OH). 6× Myc ER81^(2–477)^ and 6× Myc-ER81^(249–477)^ were kindly provided by Dr. Ralf Janknecht (Mayo Clinic, Rochester, MN). Human VE-cadherin cDNA was kindly provided by Dr. William A. Miller (Cornell University, New York, NY).

### Cell Culture and Transfection

Early passage (<passage 30) SKBR-3 cells were maintained in Dulbecco's Modified Eagle's Medium (DMEM) supplemented with 10% fetal bovine serum (FBS) in 5% CO_2_ incubator at 37°C. All transient transfection experiments of plasmid DNA and siRNA were performed with Amaxa electroporation system (Amaxa, Inc, Gaithersburg, MD) according to the manufacturer's protocol.

### Electron Microscopy

Following 48 h of RA treatment 80% confluent SkBr3 cells were rinsed in PBS and trypsinized for 30 seconds to detach cell sheets from the plate but maintain cell-cell contacts. 10% FBS was added to stop the action of the trypsin. Cells were gently centrifuged (300×g) for 2 minutes, the medium decanted and one ml of warm 2% liquid agar was added to the cell pellet and allowed to solidify (5 minutes). The cells were fixed in 2% glutataldehyde for three hours at room temperature rinsed in PBS and post fixed in 2% Osmium Tetroxide overnight at 4°C. The pellets were rinsed again in PBS, dehydrated and embedded in epon 812. 600 angstrom sections were cut and viewed on a Siemens transmission electron microscope.

### Matrigel Assay

Each well of a 12-well glass-bottom dish (MatTek, Ashland, MA) was coated with 100 µL of Matrigel (BD Biosciences, San Jose, CA) and incubated 15 min at 37°C. SKBR-3 cells (15,000 cells/100 µl medium) were gently plated on top of the Matrigel layer directly and further incubated 30 min at 37°C. One milliliter of growth medium was added along with either ethanol control or the appropriate concentration of 9-*cis*-RA. Cells were maintained at 37°C in a 5% CO_2_ incubator, and the media and 9-*cis*-RA were replenished every 48 h. On day 11, cells were stained with 1 µg/ml of Calcein AM (Invitrogen) for 30 min at 37°C before visualization.

### Network Formation and Mixed Vessel Assay

SKBR-3 cells were pre-treated for 24 hours with ethanol control or 10^−7^M 9-*cis*-retinoic acid. The SKBR-3 cells were trypsinized, and cell suspended to 10^6^cells/ml. Glass-bottom dishes (Mattek) were plated with 125 ul of Matrigel (BD Biosciences) and allowed to polymerize for 30 min in the incubator. 100 ul of cell suspension was plated on top of the Matrigel layer and incubated at 37°C for 30 minutes. 1 mL of DMEM (Invitrogen) or EGM (Cambrex) supplemented with control or 10^−7^M RA was added to the appropriate wells. For mixed vessel assays SKBR-3 cells and HUVEC cells were treated with 15 µM CFMDA (Invitrogen) or 15 µM CMTPX (Invitrogen), respectively for 30 minutes at 37°C. The media was changed and cells were incubated for an additional 30 minutes at 37°C. The SKBR-3 cells and HUVECs were trypsinized, and suspended to 5×10^5^cells/ml. 50 ul of HUVECs were mixed with an equal volume of treated or untreated SKBR-3 cells. 100 ul of each suspension was plated on top of the Matrigel layer and incubated at 37°C for 30 minutes. 1 mL of DMEM (Invitrogen) or EGM (Cambrex) supplemented with control or 10^−7^M RA was added to the appropriate wells. The networks and mixed vessels were imaged using an Olympus IX71 Inverted Fluorescent Microscope.

### Microarray Analysis

SKBR-3 cells were incubated in the presence or absence of 9-*cis*-RA (1 µM) for 48 h. Total RNA was isolated using Trizol (Invitrogen) combined with RNAeasy (Qiagen, Valencia, CA) and was amplified according to the Affymetrix protocol (GeneChip Eukaryotic Small Sample target labeling Assay Version II) modified so that the ethanol precipitation cDNA cleanup step was substituted by Qiaquick PCR purification kit (Qiagen). Biotin-11-CTP and biotin-16-UTP (Enzo Diagnostics, Farmingdale, NY) was incorporated during in vitro transcription and 20 ug of the biotinylated cRNA product was fragmented at 94°C for 25 min. Treated and untreated samples were amplified, labeled, fragmented and hybridized in the same run. Hybridizations to Affymetrix HG-U133A GeneChips were performed at 45°C for 16 h followed by staining and washing as described in the manufacturer's instructions. The processed chips were then scanned using an Affimatrix GeneArray scanner. Grid alignment and raw data generation were performed using Affymetrix GeneChip 5.0 Software. For quality control, oligo B2 was hybridized to the chip and the checkerboard pattern in each corner of the chip analyzed. BioB, bioC and bioD probes are added to each sample to standardize hybridization, staining and washing procedures. Raw expression values representing the average difference in hybridization intensity between oligonucleotides containing single base pair mismatches, was measured.

### Real-time quantitative PCR

Relative quantification was used to evaluate the raw data obtained from real-time PCR (7900 HT real time PCR system, 96 well format, Applied Biosystems, Foster City, CA). All standards and unknowns were performed in triplicate. The average value of the triplicate readings for each unknown was then divided by the corresponding value for 18S ribosomal RNA to normalize the data. After normalization, the value obtained for the treated unknown was divided by the value obtained for the corresponding untreated sample. The final value obtained was a measure of the fold change in gene expression for the particular genes of interest between the treated sample and the untreated sample. For all analyses a *p*-value of <0.05 was considered to be statistically significant.

### Reverse transcriptase PCR

Total RNA was isolated with Trizol reagent (Invitrogen). cDNA was prepared by first strand cDNA synthesis kit (Invitrogen). PCR primer sequences were as followings. ER81-F (5′-CCAAACTCAACTCATACACCGAAACC-3′), ER81-R (5′-TGGCTCTTGTTTGATGTCTCC-3′), E-selectin-F (5′-CCTACAAGTCCTCTTGTGCC-3′), E-selectin-R (5′-GCTAATGTCAGGAGGGAGAG-3′), cyclooxygenase (Cox)-1-F (5′-AGGAGTACAGCTACGAGCAG-3′), Cox-1-R (5′-CCTCAGAGCTCTGTGGATGG-3′), β-actin-F (5′-TGACGGGGTCACCCACACTGTGCCCATCTA-3′), β-actin-R (5′-CTAGAAGCATTTG CGGTGGACGATGGAGGG-3′).

### Immunocytochemistry

SKBR-3 cells seeded on glass cover slips in DMEM medium (10^5^ cells/well in 24 well plate) were treated with either 9-*cis*-RA (0.1 or 1 µM) or control medium and incubated for indicated times. Then cells were fixed with cold methanol at −20°C for 20 min for VE-cadherin staining, or with freshly prepared 3.7% formaldehyde for 15 min at room temperature (RT) followed by permeablization with 0.1% Triton X-100/PBS for 5 min for phalloidin staining. After blocking with 5% BSA/PBS for 1 h at RT, fixed cells were incubated overnight at 4°C with VE-cadherin antibody diluted 1∶1000 (mouse anti-VE-cadherin) or 1∶200 (goat anti-VE-cadherin) in 2% BSA/PBS, followed by incubation with Alexa Fluor 488-labeled anti-mouse IgG (1∶1000, Invitrogen) or anti-goat IgG (1∶1000, Invitrogen) for 30 min at RT. For phalloidin staining, Alexa Fluor 488 or 568-labeled phalloidin was used (1∶2000, Invitrogen). For nuclear staining, 4′, 6-Diamidine-2′-phenylindole dihydrochloride (DAPI) was used. Nikon E600 Fluorescence Microscope with Hamamatsu Orca-100 and 20×, 40×, 60× objective lens and MetaMorph (version 6.1.5) imaging analysis software (Universal Imaging Corp.) were used to detect fluorescence. Images were processed with Adobe Photoshop Elements 2.0 (Adobe Inc., San Jose, CA).

### Immunoblotting

Eighty-90% confluent SKBR-3 cells were incubated with DMEM in the presence or absence of 9-*cis*-RA for indicated times. Cells were rinsed twice with PBS and lysed with buffer containing 1% NP-40, 1% sodium deoxycholate, 0.1% SDS, 150 mM NaCl, 10 mM sodium phosphate, pH 7.2 and complete mini protease inhibitors (Roche Applied Science, Indianapolis, IN). Cell lysates were clarified by centrifugation at 14,000 rpm for 10 min at 4°C. Protein concentration was determined with a Bio-Rad DC reagent (Bio-Rad, Hercules, CA). After SDS-PAGE, proteins were transferred to Immobilon P (Millipore, Billerica, MA). Membranes were blocked with 5% milk in Tris-Buffered Saline containing 0.1% Tween-20, and incubated with primary antibody overnight at 4°C and subsequently with HRP-labeled secondary antibody. Proteins were visualized with ECL reagents (Amersham Biosciences, Piscataway, NJ) or SuperSignal West Femto (Pierce biotechnology Inc., Rockford, IL), using X-ray films (Denville Scientific Inc., Metuchen, NJ).

### Electrophoretic Mobility Shift Assays (EMSA)

#### Nuclear extract preparation

SKBR-3 cells were incubated in the presence or absence of 9-*cis*-RA (1 µM) for 48 h, and washed twice with PBS, harvested with ice-cold PBS containing protease/phosphatase inhibitors (1 mM sodium orthovanadate, 10 mM β-glycerophosphate, 1 mM DTT [Dithiothreitol], 1 mM PMSF [Phenylmethylsulfonyl Fluoride]). Cells were spun down at 1,200 rpm for 5 min at 4°C, and pellets were resuspended with ice-cold Buffer A (10 mM HEPES, pH 7.9, 10 mM KCl, 0.1 mM EDTA, 0.1 mM EGTA) containing protease/phosphatase inhibitors. After incubation on ice for 10–15 min, Triton-X 100 was added (0.5%), and cells were vortexed for 10 sec, spun down at 3,000 rpm for 3 min at RT, and supernatant (cytoplasmic extract) was removed. The pellet was re-suspended with Buffer C (20 mM HEPES, pH 7.9, 400 mM NaCl, 1 mM EDTA, 1 mM EGTA) containing protease/phosphatase inhibitors, vortexed, incubated on ice for 15 min, and spun down for 5 min at 12,000 rpm at 4°C. The supernatant was used as nuclear extract.

#### ^32^P-DNA oligonucleotide probes

Sense and antisense strand oligo DNA were separately dissolved in TE (pH 8.0). Denaturing and annealing were performed in annealing buffer (10 mM Tris-HCl pH 7,5, 50 mM NaCl, 1 mM EDTA). 5′-end phosphorylation of the annealed DNA was performed using T4 polynucleotide kinase (New England BioLabs, Beverly, MA) and γ-^32^P-ATP (Amersham) at 37°C for 10 min. Labeled probes were purified using G-25 spin columns.

#### Binding reaction and electrophoresis

Nuclear extract (2 µg) was incubated with 5× binding buffer (Promega, Cat# E3581) for 10–15 min at RT. Ten fmol of DNA probe was added and the mixture was incubated for 15–20 min at RT. In some experiments, antibody was added to the mixture, and further incubated for 10 min at RT. Loading buffer (3.3% Ficoll 400, 1.67% Glycerol, 0.041% Orange G) was added to each sample, and electrophoresed with 6% TBE/polyacrylamide gel and 0.5× TBE buffer. Gels were fixed with 40% methanol and 10% acetic acid for 15 min at RT, and dried under vacuum for 2 h at 80°C. Gels were scanned by a Molecular Dynamics 445 SI Phosphorimager, and analyzed with ImageQuaNT software (Amersham Biosciences, Piscataway, NJ).

The following probes were used:

**Table:** 

WT	: CAAAGGAACAATAACAGGAAACCATCCCAG
SOX-9 MUTANT	: CAAAGG**T**AC**T**ATAACAGGAAACCATCCCAG
5′ ETV MUTANT	: AA**CT**AACAATAACAGGAAACCATCCCAG
3′ ETV MUTANT	: CAAAGGAACAATAACAGG**CT**ACCATCCCAG
5′&3′ ETV MUTANT	: AA**CT**AACAATAACAGG**CT**ACCATCCCAG
ALL MUTANT	: AA**CTT**AC**T**ATAACAGG**CT**ACCATCCCAG

### Antibody Neutralization

Mouse monoclonal antibody raised against an antigen encoding the extracellular domain of human VE-cadherin (amino acids 48–593) was used as a neutralizing antibody (R&D systems). Cells were pre-treated with either the VE-cadherin antibody or with control mouse IgG (50 µg/ml) for 6 h, followed by 48 h incubation in the presence or absence of 9-*cis*-RA (0.1 µM).

## Results

### 9-*cis*-RA induced an endothelial-like morphological change in SKBR-3 cells

**Figure 1:**
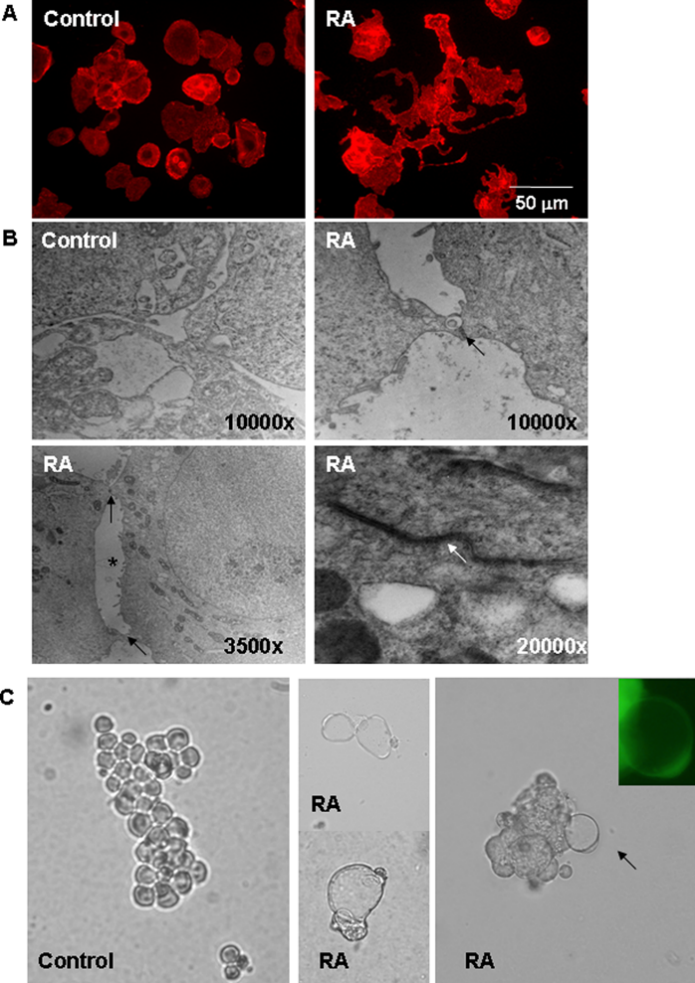
Morphological changes in SKBR-3 cells induced by 9-*cis*-RA treatment. (A) Phalloidin staining. Untreated SKBR-3 cells exhibited cuboidal shape. After 48 h treatment with 9-*cis*-RA (1 µM), SKBR-3 cells became flattened, enlarged and showed increased cell-cell adhesion, and often extended a rim of cytoplasm to form lumen-like structures. (B) Electron Microscopy. SKBR-3 cells treated with 9-*cis*-RA showed cell-cell junctions (black arrow) and lumens (white arrow), while untreated cells did not form these structures. (C) Matrigel assays at 1.5×10^5^cells/ml. Control SKBR-3 cells (left), SKBR-3 cells treated with 10^−9 ^M 9-*cis*-RA for 9 days (middle) and at day 11 (right). Green image in Inset indicates calcein staining.

**Figure 2:**
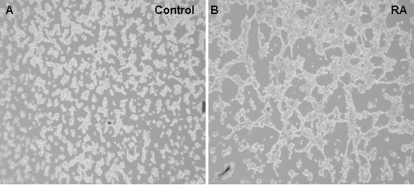
9-*cis*-RA induces SKBR3 cells to form networks in Matrigel. Matrigel assays at 1×10^6^cells/ml. (A) SKBR3 cells grown in Matrigel for 48 h. (B) SKBR3 cells pretreated for 24 h with 10^−7^M RA and grown in Matrigel for 48 h.

SKBR-3 cells treated with 9-*cis*-RA for 48 h became flattened and/or extended (Fig. 1A) in compared with control. In many cells, actin-lined lumen-like structures as well as cytoplasmic extensions, which resembled channels, sinuses, and vessel-like structures reminiscent of differentiated endothelial cell cultures were observed (Fig. 1A, right). Electron microscopy further revealed formation of cell-cell junctions in the presence of 9-*cis*-RA, indicating formation of rudimentary lumen structure in SKBR-3 cells (Fig. 1B). When cells were grown at a density of 1.5×10^5^cells/ml in Matrigel, control cells formed grape-like clusters after 9 days (Fig. 1C, left). In contrast, cells growing in Matrigel and treated with concentrations of 9-*cis-*RA less than10^−7^ M, often exhibited sinus-like structures (Fig. 1C, middle and right). Cells treated with 10^−9 ^M 9-*cis*-RA for 9 days formed colonies of fused cells with sinus-like structures (Fig. 1C, middle). The cytoplasmic extensions seen at day 11 contained live cells as indicated by calcein staining (Fig. 1C, right, inset). When pre-treated for 24 h with 10^−7^ M 9-*cis-*RA and grown at a density of 0.5–1×10^6^cells/ml SKBR3 cells formed extensive network structures in Matrigel (Figure 2).

Microarray analysis revealed that many “endothelial” genes were induced by 9-*cis*-RA in SKBR-3 cells. Principal component analysis illustrated the excellent reproducibility of our microarray data (Fig. S1A). Table 1 shows selected “endothelial-specific” genes that were up-regulated by 9-*cis*-RA. These included Cox-1, ets family member ER81, VE-cadherin, tissue factor pathway inhibitor 2 and E-selectin. These data indicated that the “epithelial” differentiation we observed in our earlier studies is more likely an “endothelial-like” differentiation [14]. However not all endothelial genes are regulated by RA, for example neither von Willebrands factor nor N-cadherin were induced by RA (not shown). Real-time PCR analysis demonstrated that 9-*cis*-RA treatment induced VE-cadherin mRNA in not only SKBR-3, but also T47D, MCF-7 and BT474 breast cancer cell lines (Fig. S1B). Other genes, such as Sox-4, Sox-18 and Sox-9, which are not known to be associated with endothelial differentiation, were also reproducibly elevated following 48 h of RA treatment (Table 1).

**Table 1:** Endothelial genes significantly (*p*<0.01) induced by 9-*cis* RA in SKBR-3 cells.

Gene Name	Gene ID# (Entrez Gene)	Accession # (GenBank)	Fold induction
Prostaglandin endoperoxide synthase 1 (Cox-1)	5742	M59979.1	16
ETS variant gene 1 (ETV1; ER81)	2115	NM_004956.3	11.8
Tissue factor pathway inhibitor-2	7980	L27624.1	11.9
VE-cadherin (cadherin-5)	1003	X79981.1	11.4
E-selectin	6401	M30640.1	10.5
Lipocalin	3933	NM_002297.2	5.9
Bradykinin receptor B2	624	S56772.1	5.5
Sox-18*	54345	AB033888.1	5.4
Fibrinogen beta chain	2244	NM_005141.2	3.9
Sox-4*	6659	AF070669.1	3.6
Ets-like factor 5	2001	AF049703.1	3
Sox-9*	6662	S74506.1	3
Fibrinogen gamma chain	2266	NM_021870.2	2.8
Endothelin converting enzyme	1889	D49471.1	2.6
Endothelin-2	1907	M65199.1	2.4
Vascular endothelial growth factor D (VEGF-D)	2277	AJ000185.1	1.8
Endothelial differentiation G-protein coupled receptor	1901	M31210.1	1.7
Endothelial PAS domain protein	2034	U81984.1	1.7

* sox genes not endothelial specific.

### 9-*cis*-RA treatment resulted in a rapid and sustained increase in VE-cadherin expression in SKBR-3 cells

**Figure 3:**
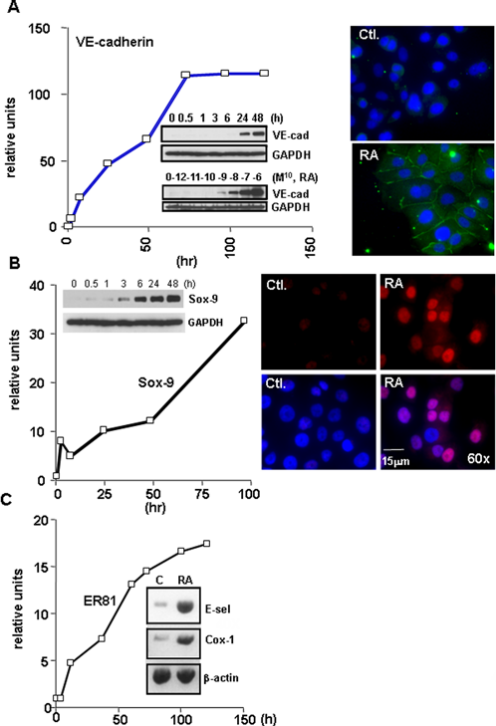
9-*cis*-RA induced VE-cadherin, Sox-9 and ER81 expression. (A) VE-cadherin induction by 9-*cis*-RA. Bar graph indicates VE-cadherin real-time PCR in SKBR-3 cells treated with 9-*cis*-RA (1 µM) for indicated times. Insets are Western blots showing time (upper)- and dose (lower)- dependence of 9-*cis*-RA on VE-cadherin protein expression. GAPDH was used as a loading control. Right panel shows 9-*cis*-RA (0.1 µM) induced expression and the membrane localization of VE-cadherin (green) in SKBR-3 cells after 48 h treatment. Blue staining represents nuclei stained with DAPI. (B) Sox-9 induction by 9-*cis*-RA. Bar graph indicates Sox-9 real-time PCR. Inset shows time-dependent induction of Sox-9 protein. Right panel shows immunostaining. 9-*cis*-RA-induced Sox-9 (red) was localized at nuclei in SKBR-3 cells. (C) ER81 induction by 9-*cis*-RA. Bar graph indicates ER81 real-time PCR. (D) Reverse transcriptase-PCR showing induction of E-selectin and Cox-1 by 9-*cis*-RA (0.1 µM, 48 h). β-actin was used as an internal control. All of these data is representative result of more than three independent experiments.

Among the many endothelial genes identified by microarray analysis, VE-cadherin is known to play a major role in angiogenesis [15]. Using real-time PCR, we confirmed that the level of VE-cadherin transcripts was increased in response to 9-*cis*-RA (Fig. 3A). Steady state levels of VE-cadherin transcripts were significantly elevated within 2 h of 9-*cis*-RA treatment (6.84 fold), and were sustained for at least 120 h (maximum 116 fold). We also examined the time- and dose-dependent effects of 9-*cis*-RA on VE-cadherin protein (Fig. 3A, inset). Robust induction of VE-cadherin protein was detected at 24 h after treatment with 9-*cis*-RA. In addition, concentrations as low as 10^−9^ M 9-*cis*-RA resulted in significant increases in VE-cadherin protein after 48 h, indicating that 9-*cis*-RA is able to induce VE-cadherin in SKBR-3 cells at levels below the physiological concentration (10^−8^ M). Immunostaining analysis also confirmed that 9-*cis*-RA induces endogenous VE-cadherin which is primarily localized at the cell membrane, resulting in cell-cell adhesion in SKBR-3 cells (Fig. 3A, right).

### 9-*cis*-RA rapidly and significantly induced Sox-9 and ER81

Our *in silico* studies indicated that VE-cadherin may not be a direct target of RA, since we were unable to find retinoic acid response elements (RAREs) in the human VE-cadherin promoter. Therefore, we hypothesized that the effects of RA on VE-cadherin expression are mediated by RA induction of other, perhaps known, endothelial regulatory genes. VE-cadherin expression is known to be regulated by members of the ets family of transcription factors [16], [17]. Indeed, our examination of the VE-cadherin promoter suggested ets and sox binding sites in VE-cadherin promoter. We reasoned that any candidate mediator genes for VE-cadherin expression would be also rapidly induced by 9-*cis*-RA in SKBR-3 cells. Sox-9, a member of the high mobility group (HMG) box gene family of transcription factors, was one of the candidate genes identified in our microarray. Indeed, Sox-9 is known as a target of RA. In the Sox-9 promoter, there are three potential RARE sites at 500 bp upstream from the transcription initiation site [18]. Another candidate gene was ER81 (ETV1; ets variant gene1). The ER81 promoter contains 4 RARE sites, and RA induces Ets1 transcription. Using real-time PCR analysis We found that he Sox-9 transcript was rapidly elevated by 9-*cis*-RA-treatment (Fig. 3B, left), and was increased 8.28 fold after 2 h exposure to 9-*cis*-RA. Significant induction of Sox-9 protein was also observed, and it preceded the expression of VE-cadherin (Fig. 3B, left inset). Induction of Sox-4 and Sox-18 by 9-*cis*-RA were also observed, but levels did not begin to increase until well after VE-cadherin induction (not shown). Increase of nuclear expression of Sox-9 by 9-*cis*-RA was also observed by immunostaining (Fig. 3B right). Similarly, we confirmed that the transcript of ER81 increased more than two fold following 2 h of 9-*cis*-RA treatment (Fig. 3C). Likewise, 9-*cis*-RA-induction of Cox-1 and E-selectin were also detected by conventional reverse transcriptase-PCR (Fig. 3C inset).

### Sox-9 and ER81 were both necessary for 9-*cis*-RA-mediated VE-cadherin expression, but neither was sufficient to induce VE-cadherin in SKBR-3 cells

**Figure 4:**
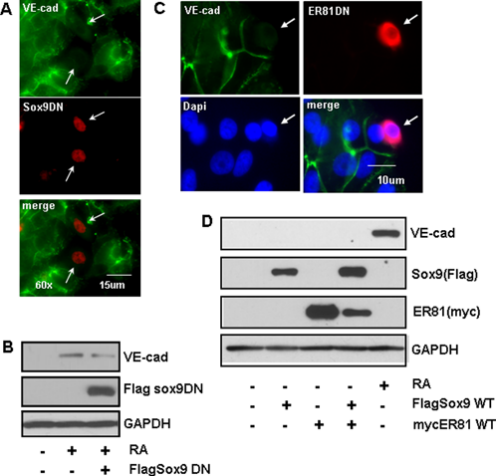
Sox-9 and ER81 were necessary for RA-mediated VE-cadherin expression, but not sufficient to induce VE-cadherin. (A) VE-cadherin expression lacked in SKBR-3 cells expressing DN form of Sox-9. SKBR-3 cells were transfected with FLAG-Sox-9 DN by Amaxa transfection, and treated with 9-*cis*-RA (0.1 µM). After 48 h, cells were fixed and double-stained with anti-FLAG (red) and anti-VE-cadherin (green) antibodies. The pictures show representative result from multiple experimental samples. (B) Western blot analysis. RA-induced VE-cadherin expression was partially inhibited by FLAG-Sox-9 DN. (C) VE-cadherin expression lacked in SKBR-3 cells expressing DN form of ER81. SKBR-3 cells were transfected with 6xMyc-ER81 DN by Amaxa transfection. Cells were stained with anti-Myc (red), anti-VE-cadherin (green) antibodies and DAPI (blue). The pictures show representative result from multiple experimental samples. (D) The effect of transient transfection of FLAG-Sox-9 WT and 6xMyc-ER81 WT in SKBR-3 cells. VE-cadherin expression was not induced by FLAG-Sox-9 WT or Myc-ER81 WT when expressed independently or together. Data is representative of three independent experiments.

Because Sox-9 was rapidly induced by 9-*cis*-RA (Fig. 3B) and the VE-cadherin promoter contains a well-conserved Sox-9 binding site, we considered Sox-9 as a candidate to co-operate with ER81 in the transcriptional regulation of VE-cadherin. Sox-9 is not known to be involved in endothelial differentiation, but is an important determinant of the chondrocyte lineage [19]. We next tested whether Sox-9 and ER81 mediate 9-*cis*-RA-induced VE-cadherin expression in the SKBR-3 cells. When cells were transiently transfected with DN-FLAG-Sox-9 lacking the C-terminal trans-activation domain, VE-cadherin expression was not observed in any transfected cells in the presence of 9-*cis*-RA (Fig. 4A, indicated by arrows). Efficiency of plasmid DNA transfection in SKBR-3 cells was 30–50% using Amaxa electroporation. Consistent with this, DN-FLAG-Sox-9 reduced 9-*cis*-RA-mediated induction of VE-cadherin by approximately 50% as judged by Western blot (Fig. 4B). These data suggest that Sox-9 positively mediates 9-*cis*-RA-induced VE-cadherin expression in SKBR-3 cells.

Ets transcription factors are known to be involved in angiogenesis [20]. There are Ets binding sites in the VE-cadherin promoter, and Ets1 has can positively regulate VE-cadherin transcription in endothelial cells [16], [21], [22]. Consistent with these studies, we observed that cells transfected with a DN form of ER81, lacking the N-terminal trans-activation domain, clearly lacked membrane staining of VE-cadherin (Fig. 4C).

Next we examined whether Sox-9 and ER81 are sufficient to induce VE-cadherin expression in the absence of 9-*cis*-RA. As shown in Fig. 4D, expression of WT-Sox-9, WT-ER81 or both failed to induce the expression of VE-cadherin. Taken together, these results suggested that both Sox-9 and ER81 are required for 9-*cis*-RA-dependent VE-cadherin expression in SKBR-3 cells, however, they are not sufficient to induce VE-cadherin protein in the absence of 9*-cis-*RA.

### Sox-9 and ER81 bound to the VE-cadherin promoter in response to 9-*cis*-RA

**Figure 5:**
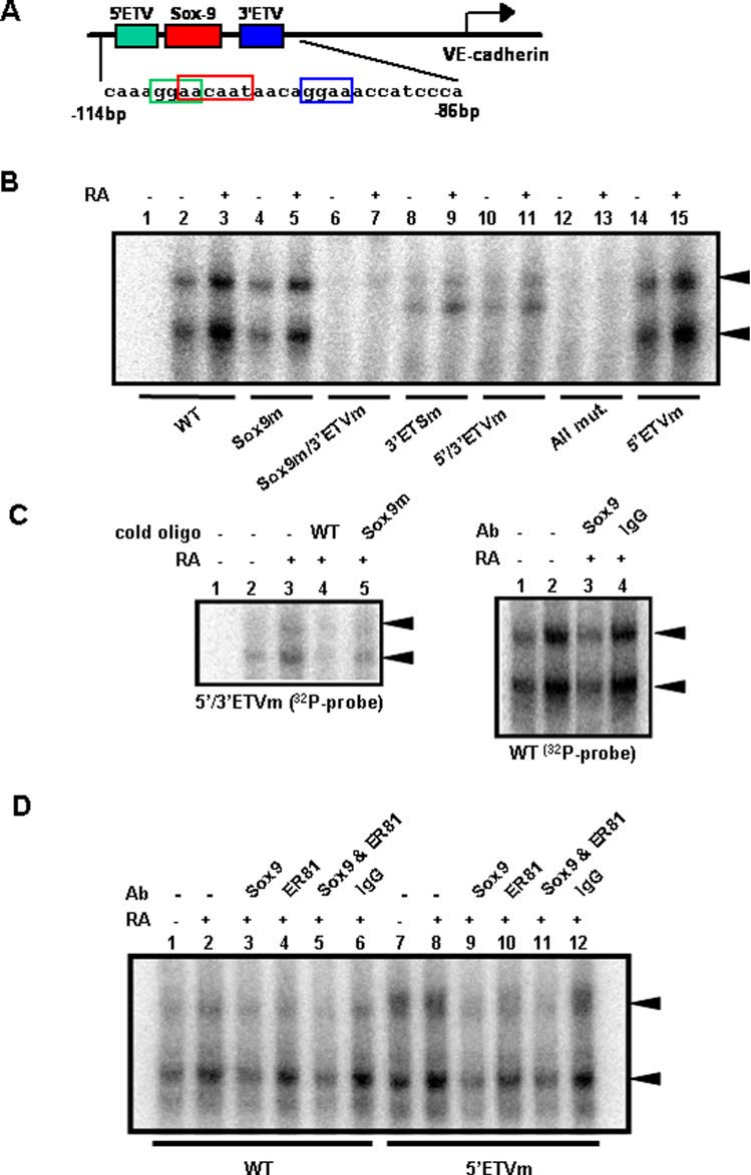
Sox-9 and ER81 bound to the human VE-cadherin promoter. (A) Sox-9 (5′-AACAAT-3′) and two ER81 (5′-GGAA-3′) binding sites were present between −114 bp and −86 bp in the human VE-cadherin gene. (B) EMSA with various ^32^P-labeled probes. With wild type probe, two major bands (arrows) were detected, and the formation of these complexes was increased by 9-*cis*-RA (1 µM). Complex formation was diminished by mutation of the Sox-9 binding site and 3′ ETV binding site but was not affected by mutation in the 5′ ETV binding site. (C) Sox-9 binds to VE-cadherin promoter: (left) competition experiment with cold WT and Sox-9 mutant probes. (right) effect of Sox-9 antibody on 9-*cis*-RA-induced DNA/protein complex formation. (D) Both Sox-9 and ER81 antibodies inhibited 9-*cis*-RA-dependent complex formation. WT probe and 5′-ETV mutant probe were used and compared. Note that 5′-ETV probe showed enhanced DNA/protein complex formation, which was reduced by either, Sox-9 antibody, ER81 antibody, and their combination. Data is representative of three independent experiments.

Sequences between −166 bp and −5 bp are critical for human VE-cadherin promoter activity in endothelial cells [16]. Our analysis revealed that the human VE-cadherin promoter has one Sox-9 binding site (5′-AACAAT-3′) and two ETV binding sites (5′-GGAA-3′) located between −114 bp and −86 bp (Fig. 5A). For convenience, we named these two ETV binding sites as **5′ ETV** site and **3′ ETV** site, which corresponds to EBS5 and EBS4, respectively, reported in previous studies [16], [22]. Using a wild type, ^32^P-labeled probe, two major DNA/protein complexes formation was observed (Fig. 5B left). Formation of these two complexes was enhanced by 9-*cis*-RA (lane 2 and 3, Fig. 5B). 5′ ETV mutant probe did not block the complex formation (lane 2/3 and lane 14/15). Sox-9 mutant alone slightly decreased the complex formation (lane 2/3 vs lane 4/5), and Sox-9 and 3′ETV double mutant failed to form the complex (lane 6/7). The effect of the double mutant was more significant compared with 3′ETV mutant alone (lane 8/9), suggesting that Sox-9 is also involved in complex formation. Thus, the Sox-9 site and the 3′ ETV site, but not the 5′ ETV site, appeared to be involved in complex formation. This finding was consistent with previous reports showing that the 3′ ETV site (EBS4) is essential for ets transcription factors to induce VE-cadherin in endothelial cells [16], [22].

With the 5′ETV/3′ETV double mutant probe, the two complexes were still observed (Fig. 5C, left, lane 2, 3). Complex formation was completely blocked by WT cold oligo (lane 4) but not Sox-9 mutant cold oligo (lane 5), suggesting that Sox-9 binding is involved in complex formation. Furthermore, DNA/protein complex formation induced by RA (Fig. 5C, right) was significantly inhibited by Sox-9 antibody (lane 3), not by rabbit IgG control (lane 4). These results indicated that Sox-9 is involved in the DNA/protein complex induced by 9-*cis*-RA in SKBR-3 cells.

Complex formation was often enhanced with the 5′ ETV mutant probe compared with WT probe (Fig. 5D; compare lane 1, and 2 and 7, 8). Complex formation induced by 5′ ETV mutant probe was significantly inhibited by ER81 antibody (Fig. 5D, compare lane 8 and 10). In addition, when both Sox-9 and ER81 antibodies were combined, the band intensity was significantly decreased (compare lane 8 and 11). The reason the 5′ ETV mutant probe enhances complex formation is not clear; however, it is possible that the 5′ ETV site might be occupied by another ets family member protein (s) which is not recognized by the ER81 antibody, therefore the blocking effect of the ER81 antibody was less significant when WT oligo probe was used (compare lane 2 and 4). Taken together, these data indicated that both Sox-9 and ER81 (at the 3′ETV site) directly bind to the VE-cadherin promoter.

### VE-cadherin antibody and VE-cadherin siRNA inhibited 9-*cis*-RA-induced morphological changes

**Figure 6:**
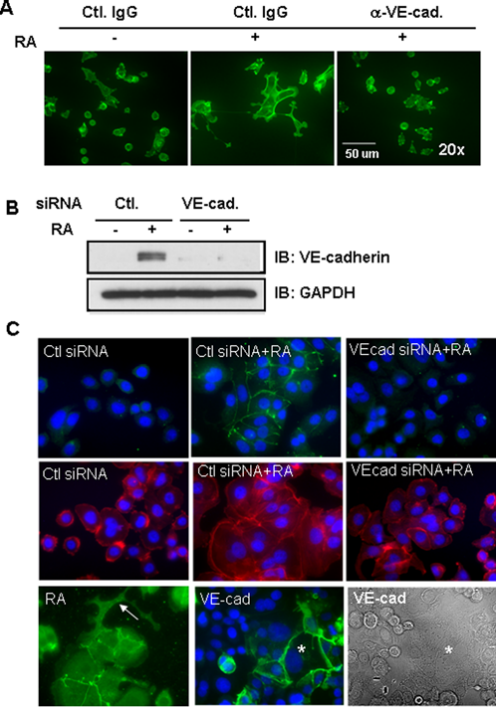
VE-cadherin played an essential role in 9-*cis*-RA-mediated morphological changes. (A) Antibody neutralization experiment. Morphology of SKBR-3 cells was visualized by phalloidin staining. (B) The effect of VE-cadherin siRNA in SKBR-3 cells. Cells were tranfected with VE-cadherin siRNA using Amaxa transfection procedure, and incubated for 6 h followed by additional 48 h incubation in the presence or absence of RA (0.1 µM). (C) The effect of VE-cadherin suppression in SKBR-3 cell shape. VE-cadherin siRNA completely blocked 9-*cis-*RA-mediated VE-cadherin expression (green) and localization to the cell surface (upper series). Phalloidin staining (red) indicated that 9-*cis*-RA-dependent, flattened morphology, cell-cell adhesion and cytoplasmic extensions were blocked by VE-cadherin siRNA (Lower series). (D) The effect of VE-cadherin transfection on cell morphology. SKBR-3 cells were transiently tranfected with human VE-cadherin using Amaxa system and analyzed 48 h after transfection. VE-cadherin expressing cells (green) were flattened (asterisk) and had more prominent cell-cell adhesion compared with non-VE-cadherin expressing cells. However, VE-cadherin expression did not stimulate the formation of cytoplasmic extension structures induced by RA (arrow). These data are representative of three independent experiments.

VE-cadherin is endothelial-specific, a major constituent of the adherens junctions, is able to protect endothelial cells from apoptosis and contributes to contact inhibition [23]. Next, we asked whether VE-cadherin plays an essential role in RA-mediated endothelial morphological changes in SKBR-3 cells. Treatment with VE-cadherin antibody raised against extracellular domain of VE-cadherin completely reversed the 9-*cis*-RA-induced morphological changes, including the formation of lumen and sinus-like structures (Fig. 6A), suggesting that homotypic interaction of VE-cadherins at the cell membrane contributes to the 9-*cis*-RA-induced cell-cell adhesion and endothelial morphological changes. We next examined the effect of VE-cadherin knockdown on 9-*cis*-RA-induced cell shape changes. Using pooled siRNA, we were able to obtain almost complete knockdown of endogenous VE-cadherin (Fig. 6B and 6C). 9-*cis*-RA-induced morphological changes were blocked in cells transfected with VE-cadherin siRNA (Fig. 6C compare middle and right panel). Thus, VE-cadherin is essential in 9-*cis*-RA-induced morphological alteration in SKBR-3 cells.

### Exogenous expression of VE-cadherin partially recapitulated 9-*cis*-RA-dependent morphological changes

We next examined if VE-cadherin expression is sufficient to reproduce the effect of 9-*cis*-RA in phenotypic changes. Human VE-cadherin was transiently tranfected into SKBR-3 cells (Fig. 6D). SKBR-3 cells expressing VE-cadherin showed prominent cell-cell adhesion (Fig. 6D, middle, asterisk) and flattened (Fig. 6D right, asterisk). However, this morphological change was not identical to that induced by 9-*cis*-RA as VE-cadherin expressing cells did not exhibit the cytoplasmic extensions observed following treatment with 9-*cis*-RA (Fig. 6C, left, arrow). These data indicated that VE-cadherin expression can partially, but not completely, reproduce endothelial-like phenotypic changes induced by 9-*cis*-RA in SKBR-3 cells.

### SKBR3 cells form mixed networks with human umbilical vein endothelial cells

**Figure 7:**
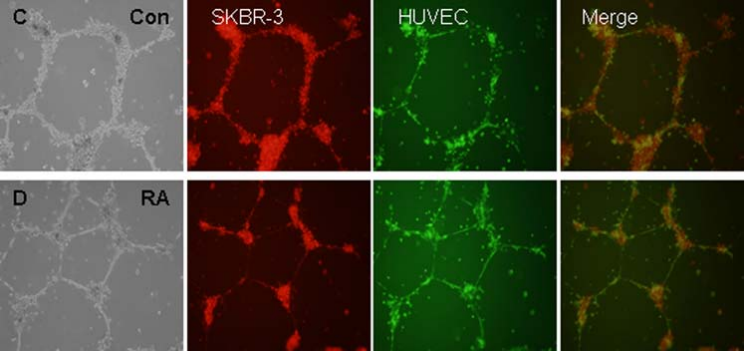
SKBR3 cells form mixed networks with human umbilical vein endothelial cells. (A) In the absence of RA SKBR3 cells decorated the surface of HUVEC networks but did not fuse or actually become part of the network. (B) In the presence of 10^−7^M RA, SKBR3 cells formed nodes from which the HUVECs either grew toward or emanated from.

Although SKBR3 cells cannot form tumors in nude mice we did find that RA-treated SKBR3 cells could form mixed vessel-like structures when co-cultured with human umbilical vein endothelial cells (HUVECs). In the absence of RA SKBR3 cells decorated the surface of HUVEC networks but did not fuse or actually become part of the network (Fig. 7A). Remarkably in the presence of RA, SKBR3 cells formed nodes from which the HUVECs either grew toward or emanated from (Figure 7B).

## Discussion

Growth, proliferation and metastasis of most tumors is dependent on adequate vasculature [24]. “Vasculogenic mimicry” is a concept that illustrates the plasticity of tumor cells, their ability to contribute to vasculogenic-like networks and the expression of genes associated with multiple cellular phenotypes. There is a growing body of *in vivo* evidence that tumor cells can line channels, sinuses and vessel-like spaces. In addition, mosaic blood vessels occur in colon carcinomas and melanomas. The formation of these networks seems to recapitulate the embryonic development of vasculogenic networks, and is associated with the distinctly patterned, extra-cellular matrix-rich networks that are observed in aggressive tumors. In aggressive melanoma with a vasculogenic phenotype, altered expression of angiogenesis/vasculogenesis-related genes such as VE-cadherin, E-selectin and tissue-factor pathway inhibitor is observed [3]. We show that 9-*cis*-RA induced these endothelial specific genes in SKBR-3 breast cancer cells with concomitant endothelial-like morphological alterations including network formation in Matrigel. SKBR3 cells cannot form tumors when explanted in nude mice so we cannot directly test if the endothelial transdifferentiation we observe influences their tumor growth. However we do find that RA-treated SKBR3 cells can form mixed vessel-like structures when co-cultured with HUVECs. These findings suggest that, under certain circumstances, RA treatment triggers an endothelial-like transdifferentiation in a subset of breast cancer cells perhaps allowing the formation of mixed vessels with the host vasculature resulting in an unfavorable clinical outcome.

VE-cadherin is exclusively expressed in endothelial cells and is restricted to cell-to-cell junctions where it mediates cell adhesion. Using VE-cadherin null embryonic body and mouse model systems, it was shown that VE-cadherin plays an essential role in angiogenesis during development [25], [26]. In addition, VE-cadherin has been implicated in tumorigenesis in the adult, and monoclonal antibodies against VE-cadherin prevent angiogenesis, suggesting that VE-cadherin is a potential target in cancer treatment [27], [28]. Our antibody blockade and siRNA experiments also showed that VE-cadherin plays a central role in RA-induced morphological changes. However, the extent to which VE-cadherin directly induces an endothelial genetic program remains elusive.

The effect of RA in the present study is reminiscent of the effects of RA in driving differentiation during development [29], [30]. In these situations, master regulatory genes, such as those in the homeobox, sox and ets families are targets for RA through RARE elements in their promoters, and these genes ultimately result in RA-induced changes in cell fate. Sox-9 plays an essential role in sex determination downstream of the sex-determining region Y (SRY) in mammals [31], [32]. In addition, Sox-9 directly regulates Col2a1, the gene encoding type II collagen , and serves as a master regulatory gene in chondrocyte differentiation [19], [33] [34], [35]. Although no previous studies have shown a role for Sox-9 in regulating VE-cadherin expression and endothelial trans-differentiation, expression of Sox-9 is detected in the developing mouse heart [36], [37], and Sox-9 null mice are embryonic lethal exhibiting major cardiovascular abnormalities [38]. These studies suggest that in addition to its role in sex determination and chondrogenesis, Sox-9 may play a broader role in the cardiovascular system. Given that VE-cadherin is widely expressed in the developing heart and vascular system , it is possible that Sox-9 is implicated in VE-cadherin expression during embryonic development [39]. Consistent with this, Sox-9 regulates the epithelial-mesenchymal transition and proliferation in endocardial cells that precedes the development of the heart valves [38].

Our data demonstrated that SKBR-3 cells treated with RA turned on an endothelial-like genetic program and acquired some phenotypic characteristics of endothelial cells such as sinus formation, lumen-like structures and the ability to form networks in Matrigel. It is possible that angiogenesis is secondary to vasculogenesis in providing a circulatory system for the growing tumor. In other words, RA-induced trans-differentiated cells may provide a homing mechanism for circulating endothelial cells (CECs) or bone marrow-derived endothelial precursor cells (EPCs). Circulating endothelial cells and progenitor cells are clearly important in tumor growth [40]. Thus, it is possible that these CECs/EPCs migrate to the tumor site and are stabilized at that location because of homotypic cell-cell interactions between cell surface antigens (e.g. VE-cadherin) that are common to both trans-differentiated tumor cells and migrating CECs/EPCs. The observations that RA-treated SKBR3 cells formed nodes form which HUVEC networks appear to nucleate is consistent with this possibility.

The ability of carcinoma cells to take on characteristics typical of cells from quite different backgrounds is well established and may be related to a stem cell-like origin. Perhaps the best example of this is the ability of breast carcinoma cells to acquire the molecular and phenotypic hallmarks of migratory and invasive mesenchymal cells [41], [42]. Our data suggests that these properties need to be taken into account when considering treatment or prevention regimens using potent differentiating agents such as vitamins A and D. Although some agents might inhibit proliferation of a subpopulation of tumor cells, they might stimulate the trans-differentiation of another subpopulation. Clearly, those tumor cells that re-acquire differentiated properties in response to such an agent still harbor the genetic defects that transformed them initially and cannot be considered as normal differentiated cells.

In summary, we demonstrated that 9-*cis*-RA induces endothelial-like trans-differentiation in SKBR-3 cells. These phenotypic changes are accompanied by significant induction of the endothelial genetic program. VE-cadherin plays a central role in these morphological alterations, and we found that both Sox-9 and ER81 bind to VE-cadherin promoter and participate in its transcriptional induction by RA. These data may explain the mixed results of clinical trials using various vitamin A analogues, and bring up the rather disturbing notion that treatments with retinoids might stimulate certain tumor cells to acquire vasculogenic properties.

## Supporting Information

Figure S1 Microarray and q-PCR analyses.
(0.26 MB DOC)
