# Supplementary material for: Role of Sox-9, ER81 and VE-Cadherin in Retinoic Acid-Mediated Trans-Differentiation of Breast Cancer Cells
Source: PLoS One. 2008 Jul 16;3(7):e2714. doi: 10.1371/journal.pone.0002714 (PMC2444023; doi:10.1371/journal.pone.0002714)
Supplement: Figure S1 — Microarray and q-PCR analyses. (0.26 MB DOC) [file pone.0002714.s001.doc]

**Supplementary Material for: Endo et al**

**Role of Sox-9, ER81 and VE-cadherin in Retinoic Acid-Mediated Trans-differentiation of Breast Cancer Cells.**

**Figure S1. 9–*cis*-RA induced the expression of endothelial-specific genes.** (A) Principal-component analysis (PCA) of triplicate microarray experiments from control and 9-*cis*-RA (1 μM)-treated SKBR-3 cells. (B) Real-time PCR amplification of VE-cadherin induction by RA in 4 breast cancer cell lines.
